# Comparing Polymerization Shrinkage Measurement Methods for Universal Shade Flowable Resin-Based Composites

**DOI:** 10.3390/biomimetics9120753

**Published:** 2024-12-11

**Authors:** Mayumi Maesako, Nicholas G. Fischer, Nagisa Matsui, Amira Elgreatly, Ahmad Mahrous, Akimasa Tsujimoto

**Affiliations:** 1 Department of Operative Dentistry, Aichi Gakuin School of Dentistry, Nagoya 464-8651, Japan; maesako@dpc.agu.ac.jp (M.M.); ag243d14@az.agu.ac.jp (N.M.); 2 Minnesota Dental Research Center for Biomaterials and Biomechanics (MDRCBB), University of Minnesota, Minneapolis, MN 55455, USA; 3 A.T. Still University Arizona School of Dentistry, Mesa, AZ 85206, USA; amiraelgreatly@atsu.edu (A.E.); ahmedmahrous@atsu.edu (A.M.); 4 Department of Operative Dentistry, University of Iowa College of Dentistry, Iowa City, IA 52242, USA; 5 Department of General Dentistry, Creighton University School of Dentistry, Omaha, NE 68102, USA

**Keywords:** polymerization shrinkage, resin composite, pycnometry, universal shade, restorative dentistry

## Abstract

Universal shade flowable composites have been introduced to mimic tooth structure with reduced color mismatch and reduced chair time and cost. However, the polymerization shrinkage of resin material may lead to sensitivity and restoration failure. The purpose of this study was to compare the polymerization shrinkage of recently introduced universal shade flowable resin-based composites using both wet and dry density methods. Using two measurement methods, ISO 17304 (wet method) and a gas displacement pycnometry system (dry method), the density of the unpolymerized and the polymerized RBCs were measured, and the polymerization shrinkage was calculated from the density difference. Scanning electron microscopy was used to visualize filler particles. The polymerization shrinkage showed significant differences between many materials. In particular, Bulk Base HARD II Medium Flow showed significantly lower polymerization shrinkage than all the other materials. Shrinkages measured by different methods were significantly different in all cases. The wet method measured a smaller shrinkage than the dry method in most cases, but the shrinkage measured for Gracefil LoFlow was larger with the wet method. Shrinkage between universal shade flowable resin-based composites significantly varied based on both material and measurement method. The polymerization shrinkage of resin-based composites is an important factor in biomimetic clinical dentistry, and work must be conducted to measure it accurately and with more standardization.

## 1. Introduction

The resin-based composite (RBC) embodies the concept of minimal intervention dentistry, which has been refined since its initial proposal by FDI in 2002, and materials have been developed by applying the results of basic research to clinical practice [1]. Research focusing on the mechanisms of adhesion between the restorative material and the tooth substrate has led to substantial improvements in bond durability [2], which has made it possible to perform restorative treatments that balance functionality and aesthetics.

**Figure 1:**
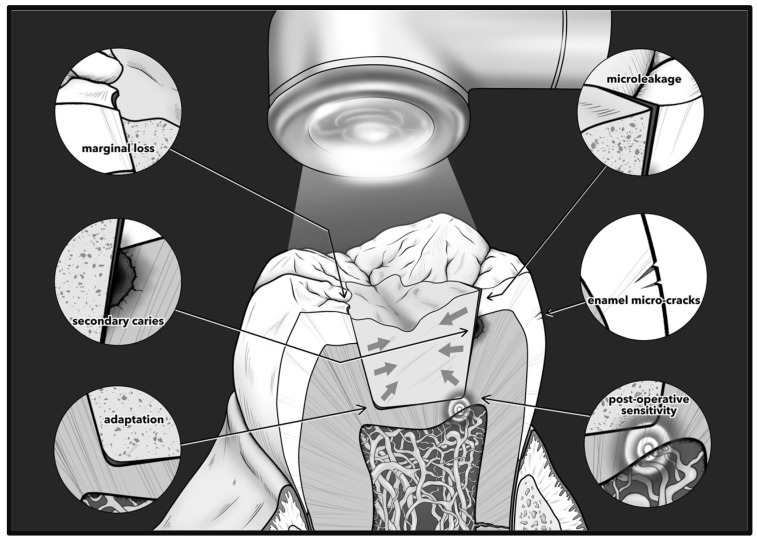
Potential consequences of polymerization shrinkage. Shrinkage may cause marginal gap formation or marginal integrity loss, microleakage, and/or enamel cracking. This may lead to secondary or recurrent caries due to bacterial infiltration and postoperative sensitivity. Poor adaptation may also result from shrinkage and thus non-ideal load distribution and possible microleakage. Figure reproduced from open access [4] with permission.

However, one of the important issues that is still a challenge to overcome in RBCs is their polymerization shrinkage [3]. Polymerization shrinkage in an RBC restoration is an unavoidable phenomenon and causes various clinical problems [4]. Potential consequences of polymerization shrinkage are summarized in Figure 1. In order to resolve this issue, various resin monomers have been developed that reduce polymerization shrinkage and disperse the stress generated by it, which is called polymerization shrinkage stress [5]. In a systematic review, Meereis et al. [6] have shown that the modification of the resin matrix with low polymerization shrinkage monomers made the largest contributions to reducing the polymerization shrinkage stress. Nevertheless, even in the latest studies, the polymerization shrinkage of RBCs has been found to be approximately 1.8–2.5% [7], and gap formation commonly occurs at the tooth/RBC interface during light polymerization in cavities with a high configuration factor (C-factor) [8]. In response, the use of layering techniques when placing RBCs in high C-factor cavities has enabled aesthetic treatments by mitigating the influence of polymerization shrinkage [4]. However, because structural flaws due to gap formation can also occur during the light polymerization of layered RBCs, bulk-fill RBCs, which allow for cavities to be filled in one increment, and universal shade RBCs, which have color created by diffraction, have been developed [9].

It is now possible to create bulk-fill universal shade RBCs. Such materials make it possible to perform aesthetic restorative treatments with color creation from the surrounding tooth structure while minimizing structural defects by placing the material in a single increment. However, concerns remain about the polymerization shrinkage and stress applied to the whole of a bulk-fill restoration during light polymerization, and the negative impact of structural defects arising from polymerization shrinkage on structural color. Therefore, it is considered important to accurately understand the polymerization shrinkages of these RBCs.

Flowable composites are versatile materials for restorative dentistry due to their low viscosity and thus enhanced flowability. They are particularly useful for use in small, conservative restorations such as small Class I, Class III, and Class V restorations wherein minimal stress-bearing or load is expected. Their excellent adaptability to preparation walls makes them ideal for marginal sealing and as liners under composite restorations to potentially minimize microleakage and postoperative sensitivity [10]. Flowable composites are also utilized in preventive procedures, such as sealants for occlusal surfaces and small pit-and-fissure caries [11]. Flowable composites are additionally beneficial in esthetics applications for repairing defects in enamel or improving the esthetics of margins in existing restorations. While they exhibit lower wear resistance and mechanical strength compared to conventional composites, their ease of handling and excellent wetting properties makes them a valuable choice for specific clinical scenarios [12].

A wet density meter has been used as described in the International Standard Organization (ISO 17304) [13] to investigate the polymerization shrinkage of RBCs [14]. This method is simple, inexpensive, and allows for the polymerization shrinkage to be measured over time. On the other hand, since the density of the RBC is measured under water before light polymerization, there is a risk that water sorption by the uncured RBC may occur during the measurement, resulting in variations in the measured values. In 1999, a dry method of measuring the density of an RBC was introduced [15], and the method has been since refined [16]. However, while two reviews of the field [17,18] found some reports of the use of gas pycnometry and reported concerns with the use of water with hydrophilic materials, they reported no direct comparisons of the two techniques. A recent paper [19] looked at the differences between two methods of measuring polymerization shrinkage stress, a closely related property to polymerization shrinkage. It found a strong correlation between measurements using the photoelastic method and measurements using the contraction forces method, but there were many cases in which the rank order of two materials differed depending on the measurement method. This shows the importance of such direct comparisons.

All methods would ideally find the same values for polymerization shrinkage for a given material. However, there are differences in the preparation methods for samples and the environment of the measurement, making agreement unlikely unless work is conducted to bring the methods into harmony. Systematic differences between two methods can, however, be taken into account, and if the methods agree in their ranking of different materials, they can both be used for comparisons of materials in which the precise values for polymerization shrinkage are not important.

Therefore, we measured the polymerization shrinkage of several recently introduced universal shade flowable RBCs using both wet and dry density methods to assess the consistency of the two methods.

## 2. Materials and Methods

The polymerization shrinkage of six resin-based composites (RBCs) was compared using two density-based techniques. The density of samples of the RBCs was measured before and after light polymerization, and the polymerization shrinkage calculated from the difference. In one technique, the density was measured by immersing the sample in aqueous solution and applying Archimedes’ principle. In the other, the density was measured by gas displacement pycnometry. The measured polymerization shrinkages were compared using two-way ANOVA and Tukey’s honest significant difference (HSD) test.

### 2.1. Study Materials

The universal shade flowable RBCs used in this study were Bulk Base HARD II Medium Flow (BBH II M; Sun Medical, Shiga, Japan), A·UNO Flow (AUF; Yamakin, Kochi, Japan), Clearfil Majesty ES Flow Low (CMEF; Kuraray Noritake Dental, Tokyo, Japan), Gracefil LoFlow (GFL; GC, Tokyo, Japan), Omnichroma Flow (OCF; Tokuyama Dental, Tokyo, Japan), and Omnichroma Flow Bulk (OCFB; Tokuyama Dental, Tokyo, Japan). Their components are shown in Table 1.

### 2.2. Polymerization Shrinkage Measurement (Wet Method)

**Figure 2:**
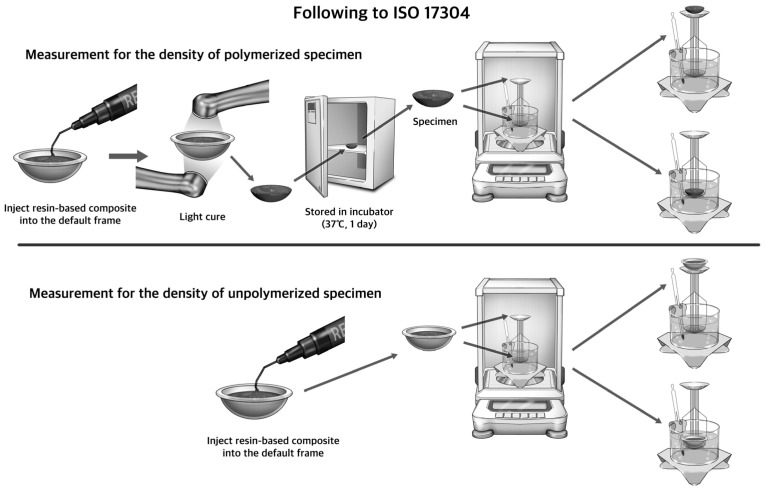
The wet method experimental procedure following ISO 17304.

The wet measurement of polymerization shrinkage of the RBCs was carried out according to ISO/FDIS 17304. The experimental procedure is shown in Figure 2. In order to measure the density of the polymerized RBCs, the flowable resin was light-irradiated for 40 s on both sides with light of 380–430 nm using a light irradiator (PenCure 2000; Morita, Kyoto, Japan) and stored in incubator (37 °C, for 1 day). The density of unpolymerized RBCs was measured by placing a quantity of unpolymerized RBCs equal to the weight of the polymerized RBC specimens into the standardized frame. Then, an electronic balance (AUY120; Shimadzu Corporation, Kyoto, Japan) equipped with a specific gravity measurement kit (SMK-401; Shimadzu Corporation, Kyoto, Japan) was used to measure the density of the unpolymerized RBCs and the polymerized RBCs, and the polymerization shrinkage was calculated from the difference in density. A 1% wt solution of sodium lauryl sulphate in water was used as the buoyancy medium. The densities of the unpolymerized and polymerized resin composites were determined by Archimedes’ principle. The polymerization shrinkage, S, was calculated from the density of the unpolymerized RBC, ρu, and the polymerized RBC, ρc, by the following formula: S (%) = (ρc − ρu)/ρc) × 100. Five samples of each material were measured, and the mean and standard deviation for the polymerization shrinkage were calculated.

### 2.3. Polymerization Shrinkage Measurement (Dry Method)

**Figure 3:**
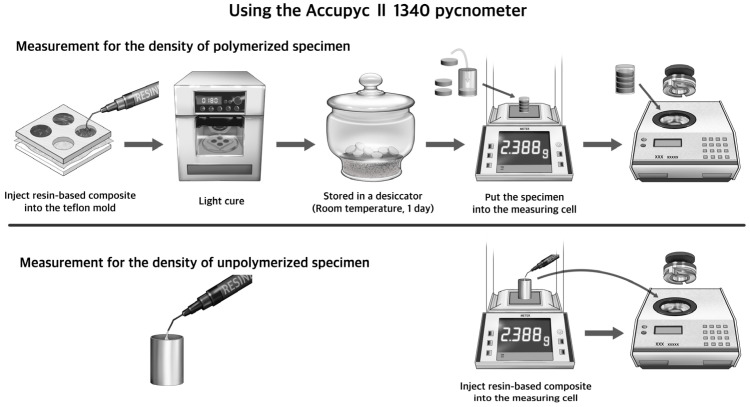
The dry method experimental procedure using the Accupyc II 1340 pycnometer.

The dry measurement of polymerization shrinkage was carried out using a gas displacement pycnometry system (AccuPyc II 1340; Micromeritics, Norcross, GA, USA). The experimental procedure is shown in Figure 3. In order to measure the density of the polymerized RBCs, specimens were prepared by placing the RBCs in a Teflon mold 15 mm in diameter and 3 mm height, and polymerizing for 180 s on both sides in a light irradiator (α Light V; Morita, Kyoto, Japan) with light of 465–475 nm. After the specimens had been stored overnight in a dessicator, four specimens were placed in a 3.5 mL cell, and the density was measured. The density of unpolymerized RBCs was measured by placing a quantity of unpolymerized RBCs equal to the weight of the four polymerized RBC specimens into the 3.5 mL cell. The polymerization shrinkage was calculated from the density of the unpolymerized RBCs, ρu, and the polymerized RBCs, ρc, by the following formula: S (%) = (ρc − ρu)/ρc) × 100. Five samples of each material were measured, and the mean and standard deviation for the polymerization shrinkage were calculated.

### 2.4. Scanning Electron Microscopy Observation

Ultrastructural observations were conducted on the polished surfaces of the RBCs using scanning electron microscopy (SEM). A custom mold, 20.0 × 10.0 mm and 4.0 mm in height, was used to form the specimens of the RBCs. The mold was placed on a glass slide covered by a matrix strip. The RBCs were placed into the mold using a condenser instrument. The top of the mold was covered with a matrix strip and the RBCs pressed with a glass slide. The exit window of an LED light curing unit was placed against the glass slide, and the RBCs were light-cured for 40 s. After photo curing, the hardened specimens were carefully removed from the mold and were polished using a gradually increasing sequence (#800, #1200, #1500, and #2000) of SiC papers in a grinder–polisher (Ecomet 3000; Buehler, Lake Bluff, IL, USA). Finally, the surfaces were polished with a soft cloth using 1.0 µm-grit diamond paste (Praxair Surface Technologies, Indianapolis, IN, USA). The samples were ultrasonically cleaned for 5 min, and the surface of each sample was sputtered with 10 nm-thick gold (MSP-1S; Vacuum Device, Ibaraki, Japan) before SEM observation. SEM observations were conducted using scanning electron microscope (SEM, VE-9800; Keyence, Osaka, Japan) with accelerating voltage of 5 kV.

### 2.5. Statistical Analysis

Data were analyzed using statistical software (IBM SPSS version 29.0.2.0, Chicago, IL, USA). After checking the normality of the data (Shapiro–Wilk test), two-way analysis of variance followed by Tukey’s HSD test at significance level α = 0.05 was performed. Pearson correlation analysis between measurements of polymerization shrinkage using the wet and the dry methods was also conducted.

## 3. Results

### 3.1. Polymerization Shrinkage Measurement

The results for the polymerization shrinkage of the resin-based composites measured by both methods are shown in Table 2. In all cases, the shrinkages measured by different methods were significantly different. In most cases, the wet method measured a smaller shrinkage, but the shrinkage measured by the wet method was larger for GFL. Pearson correlation analysis showed a statistically significant relationship between polymerization shrinkage measured with the wet and with the dry method (R = 0.832, *p* < 0.001).

Similarly, the polymerization shrinkage showed significant differences between many materials. In particular, BBH II M showed significantly lower polymerization shrinkage than all the other materials. However, by the dry measurement there was no significant difference between CMEF, OCF, and OCFB, while by the wet measurement there was no significant difference between CMEF, GFL, and OCFB. Using the dry measurement, the order of the materials was BBH II M < GFL < CMEF, OCF, OCFB < AUF, while on the wet measurement it was BBH II M < OCF < CMEF, GFL, OCFB < AUF.

The results of two-way ANOVA are shown in Table 3 and reveal that the material and measurement method both had a significant influence, and that there was a significant interaction between them.

### 3.2. SEM Observations

**Figure 4:**
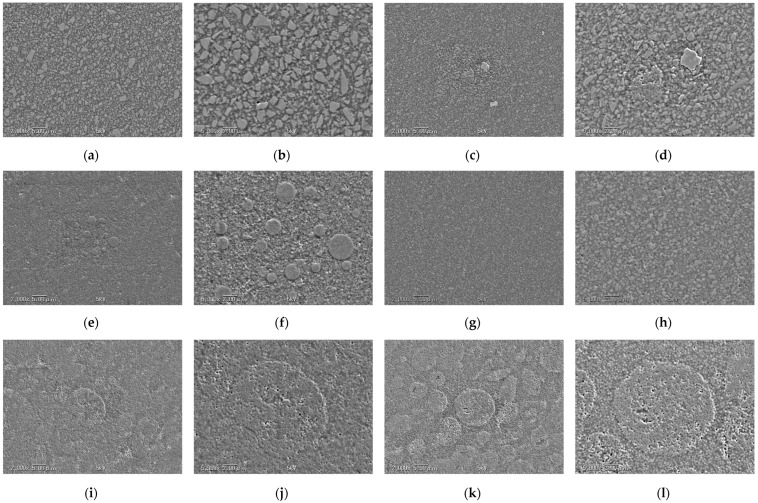
SEM micrograph of the RBC surface. (**a**): BBH II M at ×2000 magnification, (**b**): BBH II M at ×5000 magnification, (**c**): AUF at ×2000 magnification, (**d**): AUF at ×5000 magnification, (**e**): CMEF at ×2000 magnification, (**f**)**:** CMEF at ×5000 magnification, (**g**): GFL at ×2000 magnification, (**h**): GFL at ×5000 magnification (**i**): OCF at ×2000 magnification, (**j**): OCF at ×5000 magnification, (**k**): OCFB at ×2000 magnification, (**l**): OCFB at ×5000 magnification.

Figure 4 shows SEM micrographs of the RBC surface. BBH II M was observed to have irregular shaped fillers (<5 μm). In AUF, spherical nanofillers (<1 μm), fluoride-sustained release fillers (approximately 2–3 μm), and ceramics cluster fillers (approximately 5 μm) were observed. CMEF was observed to have submicron barium glass fillers and cluster fillers (approximately 1–3 μm). GFL showed irregular-shaped nanofillers (<1 μm). OCF and OCFB were observed to have 260 nm spherical inorganic fillers and 5~10 µm organic composite fillers containing the inorganic fillers. No clear differences were observed between OCF and OCFB in these observations.

## 4. Discussion

Various methods have been used to measure the polymerization shrinkage of RBCs [14], but there have been few direct comparisons. Monteiro et al. [20] compared three different methods—a coordinate measuring machine, optical coherence tomography, and the wet method—and found that they gave significantly different results. Other methods mentioned by Monteiro et al. [20] include the bonded disk method, mercury dilatometer, an optical method, gas pycnometer, strain gauges, linear displacement measurements, free linear shrinkage, and wall-to-wall shrinkage.

In the present study, we employed the method recommended by ISO [13], buoyancy (the wet method), and pycnometry (the dry method), following the specifications by Japanese Industrial Standards [21]. Our results also show significant differences between the methods, and that there was a significant interaction between the materials and the methods. This is seen most clearly in the case of GFL, which showed a larger polymerization shrinkage when measured by the wet method, while all the other materials showed a larger shrinkage with the dry method. However, the proportional change in the measured polymerization shrinkage varied between the other materials as well. The results showed small differences in the ordering of the materials between the two methods. In addition, Pearson rank correlation analysis revealed that there was a statistically significant relationship (R = 0.832, *p* < 0.001) between the values measured with the dry and with wet methods.

The differences between these two measurement methods could have implications for the interpretation of results. In particular, the dry method showed that OCF has significantly greater polymerization shrinkage than GFL, while the wet method showed that GFL has significantly greater shrinkage than OCF. These results by themselves do not indicate which method is more appropriate. However, RBCs, especially hydrophilic materials such as flowable RBCs and compomers, are known to interact with water in several ways, both physically absorbing it, and chemically reacting with it [17]. It is not easy to predict what effect this will have. Absorption of the water could lead to an increase in actual volume, but a reduction in measured volume, if the volume increase of the material is smaller than the volume of the water absorbed. Chemical reactions could influence the measured volume in either direction, depending on the nature of the reaction and its products. Thus, the wet method with a medium other than water, such as mercury, has been used for these kinds of materials. However, due to the potential for environmental mercury contamination and toxic mercury vapors, the development of alternatives is important. The dry method measures gas displacement at a constant pressure, and the gas used is helium, which is non-reactive, and so should not affect the RBCs. Thus, there are theoretical reasons to believe that the dry method may be a more reliable and consistent way to measure the polymerization shrinkage of flowable RBCs.

On the other hand, the dry method relies on measuring the change in pressure of a gas, and thus can only be used with samples that have a low vapor pressure. If the sample is emitting a significant amount of gas, that will change the pressure and make the results inaccurate. This is why the polymerized samples for gas pycnometry must be desiccated before measurement to avoid the release of water vapor. This may, however, lead to shrinkage of the sample due to drying, which may lead to a higher measured value for the polymerization shrinkage.

In this study, polymerization shrinkage ranged from 2.75% to 4.23% when measured by the wet method, and from 3.15% to 4.48% when measured by the dry method. A study of older flowable composites found a range from 2.14% to 3.73%, using the wet method [14]. The results of these studies are broadly consistent, although there were no materials in common. On the other hand, earlier studies using the dry method found polymerization shrinkage ranging from 1.14% to 1.98% [22], or from 0.56% to 3.26% [23]. These studies used conventional RBCs, rather than bulk-fill RBCs, which may explain the lower measured shrinkage. Further work is needed to develop reliable and accurate methods with which to measure the polymerization shrinkage of RBCs.

In this study, both methods showed that BBH II M had significantly lower polymerization shrinkage than the other RBCs, and the polymerization shrinkage measured by the wet method was lower than that of most of the materials investigated by Nitta et al. [14]. BBH II M is a bulk-fill RBC, designed to have reduced polymerization shrinkage, which utilizes low shrinkage monomers, which are methacrylate monomers and Bis-MPEPP, both of which have been shown to reduce polymerization shrinkage. The confirmation of this reduced polymerization shrinkage by both methods strongly suggests that this approach, namely the modification of the resin matrix in RBCs, to reducing the polymerization shrinkage is effective, which is consistent with a recent review [6]. Future research should focus on correlating polymerization stress, using methods like the photoelastic method and contraction forces method, and shrinkage itself.

An additional perspective has been advocated by some researchers focused on hygroscopic expansion or swelling from imbibement of water like that seen when resin-based materials are placed in the oral cavity. Classic work [24] showed that water imbibement partially relaxed polymerization shear stress for more hydrophobic resins and in some cases fully relieved this stress for more hydrophilic polymers. More recent work [25] demonstrated that a time frame of 2–4 weeks of storage in water released most of the contraction stress from modern resin composite formulations. The timeframe for when stress develops vs. when stress is released from water uptake remains to be studied.

Polymerization shrinkage in dental composites can be partially mitigated using various strategies aimed at reducing stress and enhancing material performance. One approach modifies the resin matrix by incorporating low-shrinkage or high-molecular-weight monomers, such as silorane-based resins, which exhibit lower volumetric contraction compared to traditional methacrylate resins [26]. Another effective method is increasing the filler content and optimizing the filler–matrix ratio. Higher filler loading can restrict shrinkage by reducing the volume of polymerizing resin [27]. Clinically, employing incremental layering techniques during restoration minimizes the volume of material polymerized at one time, thereby reducing the cumulative shrinkage stress on tooth structures [28]. Additionally, the use of other curing techniques, such as soft-start polymerization, which gradually increases the light intensity, may help control the rate of polymerization and allow for stress relaxation [29]. Research on innovative filler modifications, such as the incorporation of pre-polymerized particles, continues to advance shrinkage management to promote the longevity of dental restorations [30].

Future work should support the development of silorane-based flowable composites. As mentioned, silorane-based materials reduce polymerization shrinkage. The polymerization of silorane-based materials proceeds through a cationic-ring opening of a siloxane core with four oxirane rings attached rather than a linear chain of traditional methacrylates. This results in, for example, less than 1% total volumetric shrinkage through the compensation of the opening of the oxirane ring [26]. These monomers are also hydrophobic, which is useful to reduce staining/discoloration and water absorption. Physical and mechanical properties of silorane-based materials bonding ability are generally comparable to traditional chemistries [31]. However, despite the promising in vitro and theoretical benefits of silorane-based chemistries, evidence of the superiority of silorane-based materials is still missing from clinical trials, and they generally show similar performance to conventional materials [32].

It is generally thought that the filler content of an RBC also affects its polymerization shrinkage, as the shrinkage occurs in the resin matrix [33,34]. In theory, if the material contains a higher volume of filler, this may limit the overall shrinkage, even if the resin matrix has a relatively high shrinkage. In universal flowable RBCs, which have higher translucency and lower viscosity than conventional RBCs, the polymerization shrinkage tends to be higher than that of conventional RBCs due to lower filler content. However, the volume proportion of inorganic filler of delivered RBCs is difficult to measure. The proportion by weight can be measured by burning the material and weighing the ash, which is composed of the inorganic filler, but there is no established way to measure the proportion by volume. The SEM observations in this experiment suggest that there are differences in the volume proportion of filler between different RBCs but are not sufficiently precise to establish whether there is any relationship between those differences and differences in polymerization shrinkage. Further research is needed to establish an effective method to assess this.

## 5. Conclusions

Polymerization shrinkage measurements for universal shade flowable resin-based composites varied based on the method used but were significantly correlated. Bulk Base HARD II Medium Flow Multi showed the lowest polymerization shrinkage. Future research should propose unified standards for measuring polymerization shrinkage to better enable comparison and clinicians’ ability to select optimal materials. Future research should also consider the influence of both resin monomer and filler volume percentage on polymerization shrinkage, using density measurements to determine volumetric proportions. This may enable a better explanation of the differences observed between these materials. Furthermore, polymerization stress is more important than polymerization shrinkage in the clinic, and the stress is influenced not only by the polymerization shrinkage, but also by the mechanical properties of the material. Experiments should be performed to determine these relationships.

## Figures and Tables

**Table 1:** Composition of tested RBCs.

Materials (Code)	Compositions	Fillers	wt%	vol%	Manufacturer
Bulk Base HARD II Medium Flow Multi (BBH II M)	Bis-MPEPP Methacrylate monomers	Barium silica glass	74	54.3	Sun Medical
A·UNO Flow Basic (AUF)	UDMA Bis-GMA DEGDMA	Spherical nano filler (20–50 nm) Fluoride sustained-release filler (700 nm) Ceramics cluster filler (2–8 μm)	70	-	Yamakin
Clearfil Majesty ES Flow Low Universal (CMEF)	Hydrophobic aromatic dimethacrylate TEGDMA	Silanated barium glass filler Silanated silica filler (0.18–3.5 μm)	75	59	Kuraray Noritake Dental
Gracefil LoFlo Universal (GFL)	Bis-MEPP	Barium glass (250 nm)	69	-	GC
OMNICHROMA Flow (OCF)	UDMA 1,9-nonamethylene glycol dimethacrylate	Spherical silicazirconia filler (260 nm)	71	57	Tokuyama Dental
OMNICHROMA Flow Bulk (OCFB)	UDMA TEGDMA	Spherical silicazirconia filler (260 nm)	69	55	Tokuyama Dental

**Table 2:** Results of the two-way analysis of variance for polymerization shrinkage.

Source	SS	df	Mean Squares	F-Ratio	*p*-Value
Material	10.279	5	2.056	225.091	<0.001
Measurement method	0.771	1	0.771	84.380	<0.001
Material × Measurement method	0.767	5	0.153	16.804	<0.001

**Table 3:** Results for polymerization shrinkage.

Measurement Method	BBH II M	AUF	CMEF	GFL	OCF	OCFB
Dry	3.15 (0.04) ^aA^	4.48 (0.01) ^aB^	3.91 (0.12) ^aC^	3.47 (0.02) ^aD^	3.93 (0.03) ^aC^	3.97 (0.02) ^aC^
Wet	2.75 (0.16) ^bA^	4.23 (0.16) ^bB^	3.70 (0.04) ^bC^	3.71 (0.10) ^bC^	3.46 (0.10) ^bD^	3.70 (0.13) ^bC^

Polymerization shrinkage values (%). Mean values are presented with standard deviations in parentheses. Bulk Base HARD II Medium Flow (BBH II M), A·UNO Flow (AUF), Clearfil Majesty ES Flow Low (CMEF), Gracefil LoFlow (GFL), Omnichroma Flow (OCF), and Omnichroma Flow Bulk (OCFB). Same superscript small letter in columns indicates no significant difference (*p* > 0.05). Same superscript capital letter in rows indicates no significant difference (*p* > 0.05). Significance assessed by Tukey’s HSD test.

## Data Availability

Data are available upon request.
